# Bidirectional modulation of infralimbic dopamine D1 and D2 receptor activity regulates flexible reward seeking

**DOI:** 10.3389/fnins.2013.00126

**Published:** 2013-07-18

**Authors:** Jacqueline M. Barker, Mary M. Torregrossa, Jane R. Taylor

**Affiliations:** ^1^Department of Psychiatry, Division of Molecular Psychiatry, Center for Genes and Behavior, Yale University School of Medicine, Abraham Ribicoff Research Facilities, Connecticut Mental Health CenterNew Haven, CT, USA; ^2^Interdepartmental Neuroscience Program, Yale UniversityNew Haven, CT, USA

**Keywords:** habit, dopamine, prefrontal cortex, mouse, behavioral flexibility

## Abstract

The development of addictive behavior is marked by a loss of behavioral flexibility. In part, this is due to an increase in the ability of environmental stimuli to elicit responding and decreased importance of the action-outcome relationship in behavioral control. It has previously been demonstrated that both inactivation of and dopamine (DA) infusions in the infralimbic prefrontal cortex (PFC) can restore behavioral flexibility in paradigms measuring habitual reward seeking. Here, we investigated the mechanism by which cortical DA would act to enable goal-directed actions after the transition to habitual behavior has been established. Further, we extended this work to include a novel mouse model of compulsive-like behavior in which we assessed reward seeking despite the possibility of adverse consequences. Our data show that DA receptor D1 inhibition or D2 activation both promote the expression of a flexible responding after the development of habitual or compulsive-like behavior, and we suggest that the ability of DA infusions in the infralimbic PFC to restore sensitivity to changes in outcome value depends on activation of DA D2 receptors.

## Introduction

The transition from casual drug use to addiction is characterized by increasing loss of control over reward seeking. When a behavior is first learned, performance of the action is guided by its relationship to its outcome—*i.e*., a response is made in order to gain access to a reinforcer. Over time and after repeated execution, behavior transitions from goal-directed action to stimulus-driven habitual behavior (Dickinson, 1985). Habitual reward seeking is no longer mediated by action-outcome relationships or by a representation of the value of an outcome; rather, habitual behavior is automatically elicited by environmental cues and stimuli (c.f., Yin et al., 2008). In addition to habits, addictive behavior also involves the development of compulsive reward seeking that occurs despite adverse consequences (e.g., Everitt et al., 2008; Heyne et al., 2009). Successful treatment of addiction may require restoration of the ability to update behavior in accordance with changed contingencies and in the face of negative outcomes.

The shift in response strategy away from flexible, contingency-mediated behavior to one in which stimulus-response relationships guide behavior is paralleled by a change in the neuroanatomical substrates that mediate behavior from a prefrontal-striatal circuit in which the prefrontal cortex (PFC) monitors the action-outcome relationship, to a more dorsal circuit involving dorsolateral striatum (e.g., Yin and Knowlton, 2006; Balleine and Dickinson, 1998). However, a role for the infralimbic PFC (IL) in the expression of habitual behavior has been demonstrated. When the IL, which projects to the nucleus accumbens shell (e.g., McGeorge and Faull, 1989) and amygdala (Sesack et al., 1989), is lesioned prior to response acquisition, animals are unable to express stimulus-response habits (Killcross and Coutureau, 2003). After extended training, IL lesioned animals remain sensitive to changes in outcome value. Importantly, later research expanded on this finding to show that inactivation of the IL after extended training, at a time point where intact animals are habitual, resulted in the restoration of flexible behavior (Coutureau and Killcross, 2003). More recent work has expanded upon these findings using optogenetic manipulations to investigate online regulation of the IL in the expression of habitual behavior (Smith et al., 2012). Together, these data suggest that the IL is critically involved in the selection of response strategy in situations of conflict between automatic, habitual behaviors and flexible goal-directed actions.

Dopamine (DA) signaling within corticostriatal circuitry has been shown to play a unique role in both the formation and expression of goal-directed vs. habitual instrumental behavior (e.g., Nelson and Killcross, 2006). Our lab has shown that infusions of exogenous DA in the IL, but not the more dorsal prelimbic PFC (PL), restored sensitivity to outcome devaluation after extended training (Hitchcott et al., 2007). While a majority of these studies were performed in rats, we have found using lesion studies that the neuroanatomical mechanisms underlying habit learning are preserved in mice (Quinn et al., 2013). The mechanism by which both inactivation of and DA infusion into the IL can restore sensitivity to the action-outcome relationship is unknown in rodents. Here, we assessed the ability of DA D1 and D2-family specific manipulations in the IL to restore flexible behavior as measured by either sensitivity to changes in action-outcome contingency or reduction of compulsive-like reward-seeking behavior in mice.

## Materials and methods

### Subjects

Male C57b/6 mice were supplied from Charles River and delivered to the Yale University/Connecticut Mental Health Center mouse vivarium between 56 and 70 days of age. These mice were allowed to acclimate for 2 weeks with *ad libitum* access to food and water. All behavioral procedures were approved by the Yale University IACUC and experiments were performed in accordance with the National Institute of Health Guide for Care and Use of Laboratory Animals. After acclimation, mice were food restricted to 90–92% of free feeding weight for all experiments. They had limited access to standard chow in their homecage each day, several hours after training. The amount of food provided was adjusted to maintain weights. Homecage chow was distinct from the purified grain pellets used in both the habitual and compulsive-like food-seeking experiments. There were approximately 5–12 animals in each experimental group after exclusion of mice with inaccurate cannula placement or loss/clogging of cannula during the course of the experiments. Saline groups had large *n*'s (>12) as a cohort of control (saline) animals was included in each testing session to ensure baseline effects were consistent.

### Instrumental conditioning chambers

Instrumental chambers were identical to those described by (Barker et al., 2012). Briefly, 12 mouse instrumental chambers housed within a sound-attenuating box, were used for these experiments (Med-Associates; Georgia, VT). Each chamber was equipped with a 28 V house light located at the top of the middle panel on the left side wall, three adjacent nosepoke apertures located at the bottom of the left side wall, and a magazine located at the bottom of the middle panel on the right side wall. Grain pellets were delivered to a magazine on the opposite wall. Nosepoke apertures and reinforcement magazine were equipped with a light and photobeam sensor. A fan provided background noise and ventilation.

### Stereotaxic surgery

Mice were anesthetized using ketamine/xylazine. Bilateral cannula (Plastics One; Roanoke, VA) were implanted and mounted to the skull using standard stereotaxic techniques. Cannula were targeted to the IL at AP + 1.7, ML ± 0.25, DV-3.0 from bregma based on coordinates from Wall et al. (2004). For compulsive-like food-seeking experiments, surgeries were performed prior to any training. For instrumental habit experiments, surgeries were performed after 3 days of fixed ratio (FR) 1 training to reduce the amount of time between cannula placement and testing.

### Drugs and infusions

For tests of habitual and compulsive-like food-seeking, mice received two infusions of the same drug prior to a control and experimental session. Infusions were 0.2 uL over 2 min; internal cannula were left in place for an additional 2 min to allow for diffusion. This volume and diffusion duration were chosen based on the literature and our pilot data using thianin which suggested minimal spread to surrounding tissues at this volume and after the delayed removal of cannula. Drugs used were the D1 agonist dihydrexidine HCl (DHX; Tocris; Minneapolis, MN), D1 antagonist SCH23390 (Sigma; St. Louis, MO), D2 agonist quinpirole (Tocris) in saline, and the D2 antagonist sulpiride (Tocris) in acidified saline, each dissolved at 5 nmol per 1 ul.

### Instrumental training

During training, one nosepoke was assigned as the active nosepoke, where a response resulted in reinforcer delivery, and the others designated as inactive nosepokes. Training consisted of 1 day magazine training, 3-days fixed ratio (FR 1) training (in which each active response resulted in reinforcer delivery) and 3-days random interval (RI) 30-s training and 6 RI60 sessions. In RI sessions, reinforcement could be earned every 30 (RI30) or 60 (RI60) s on average. The actual duration of each interval was randomly determined so that reinforcement availability was not predictable. The first active response (nosepoke) after the interval ended resulted in reinforcer delivery; the duration of the next interval was then generated automatically. During each daily training session, the house light and fan were on. All sessions were 30 min in duration.

### Contingency degradation test

During degradation sessions, conditions were identical to training except that the grain pellet reinforcer was delivered on a non-contingent schedule determined by each individual animal's reinforcement rate on the day prior. Reinforcer delivery was spaced equivalently across the 30-min session. Responses on the active and inactive nosepokes were recorded, but did not result in reinforcer delivery. Infusions of drugs occurred 5 min prior to the start of the degradation session. Mice were assigned to infusion groups by matching baseline response rates, and received a 0.2 ul infusion of either saline (*n* = 17), DHX (*n* = 11), SCH23390 (*n* = 6), quinpirole (*n* = 12), or sulpiride (*n* = 9). More animals were in the saline groups as a cohort of saline animals was included with each behavioral test session to confirm baseline effects were replicated. Data were compared to a non-degraded session in which the animals received the same drug; the order of these sessions was counterbalanced and animals received one normal RI 60 training session between both test sessions where no drug was administered.

### Compulsive-like food-seeking training and test

Additionally, we assessed the effects of IL DA receptor modulation on compulsive-like behavior in mice using a modification of traditional conditioned place preference/aversion testing. Conditioning chambers were standard three chamber boxes with retractable doors (Med Associates; Georgia, VT). Chambers had distinct walls (vertical black and white stripes or diagonal marble and black stripes) and floors (wire mesh or grid). The two conditioning chambers were separated by a neutral, gray chamber. Photocell beam breaks were used to calculate time spent in each chamber, latency to enter the chamber and number of entries by Med-PC IV software. During a single habituation session, mice were placed in the neutral chamber with both doors retracted such that mice could freely explore all chambers. During conditioning, mice were confined to the “paired” chamber for 30 min with access to 30-grain pellets on days 1, 3, and 5. On days 2, 4, and 6, mice were confined to the opposite chamber for 30 min with an empty food dish.

On day 7, mice received an infusion of either a DA D1 or D2-like receptor agonist or antagonist 5 min prior to being placed in the neutral chamber with both doors retracted and were allowed to freely explore all chambers for 5 min. This duration was chosen because we were able to examine entry into both chambers and latency to enter, but no extinction was expected to occur based on our preliminary data. Mice received a 0.2ul infusion of either saline (*n* = 20), DHX (*n* = 8), SCH23390 (*n* = 7), quinpirole (*n* = 8), or sulpiride (*n* = 7). Latency to enter the chambers was the primary outcome measure.

On the following day, mice were confined to the food-paired chamber. Two minutes after placement, mice received a 2 s, 0.8 mA foot shock. Mice remained in the chamber for 60 s after the shock was terminated and were then returned to their homecage. On day 9, mice received a second infusion of the same drug as day 7. Five min after the infusion, they were returned to the gray chamber and allowed to freely enter both chambers and latency to enter the chambers was assessed in this 20 min session. Latency was selected as the primary measure of compulsive-like behavior because it was not expected to be impacted by the extinction of either the association of the chamber with footshock or the association with the food reward which may be differentially impacted by prefrontal DA manipulations. Importantly, a change in the parameters of the training conditions might have an impact on the expression of reward-seeking under conflict between reward seeking and avoidance of negative consequences, either by increasing the aversive component (e.g., through increasing the shock intensity), the value of the reward, or the extent of learning (e.g., through extended training).

### Confirmation of placement

After behavioral assessment was complete, mice were sacrificed and tissue was fixed in paraformaldehyde for confirmation of cannula placement and location of the infusion tip using standard histological techniques. If cannula were not clogged at the time of sacrifice, thianin was infused at the volume and rate used for testing (0.2 ul over 2 min). If cannula had become clogged, cannula tracts, and tips were confirmed. Mice were excluded if placement could not be confirmed to be in the IL through the use of neuroanatomical landmarks, including white matter tracts.

### Statistics

Data were analyzed with JMP Software (SAS Institute) using repeated measures analysis of variance (ANOVA). Significant interactions were further analyzed using Tukey's HSD *post-hoc* tests.

## Results

### Contingency degradation

**Figure 1:**
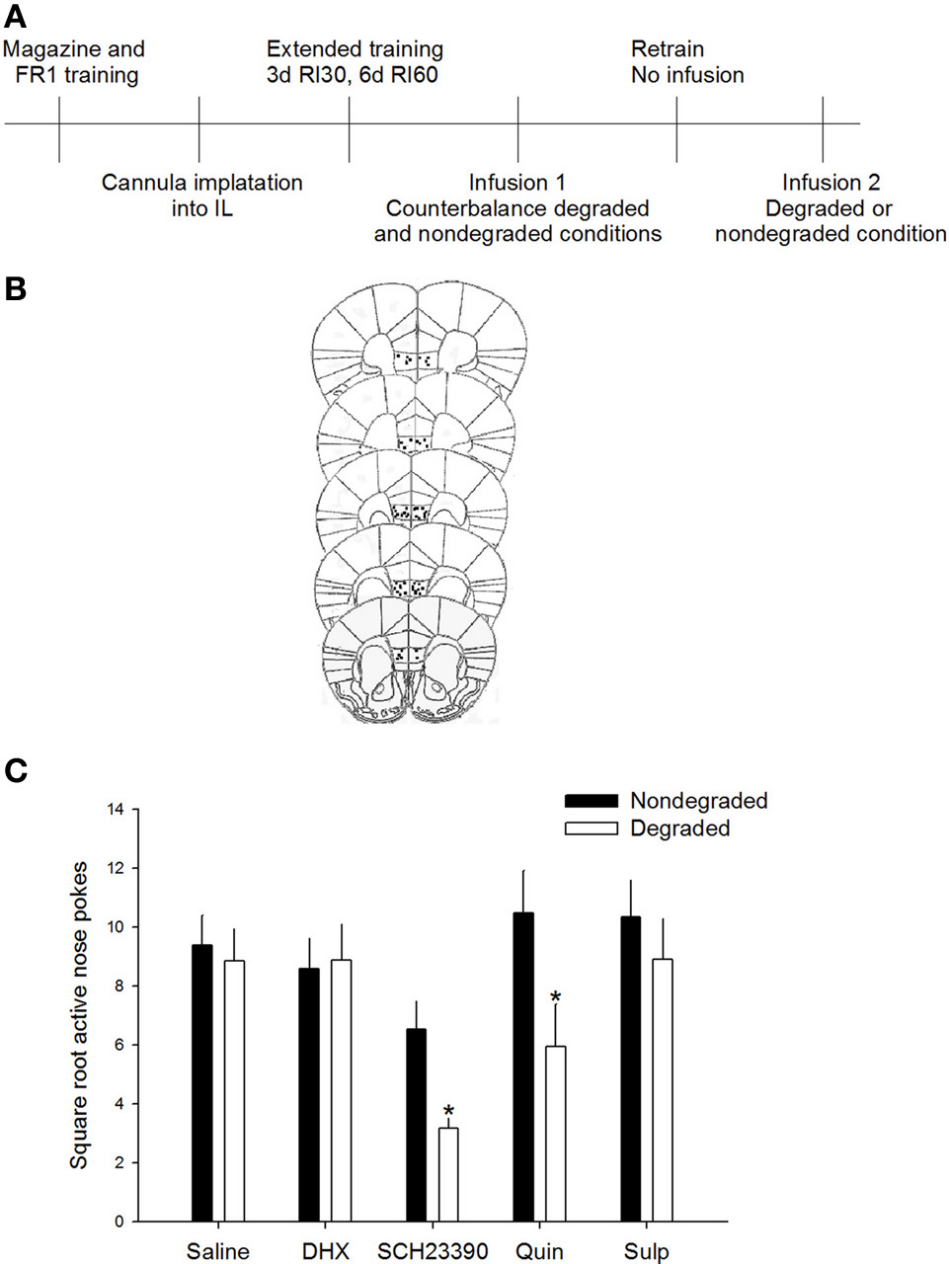
**D1 antagonism or D2 agonism restore goal-directed behavior**. **(A)** Experimental timeline. Mice only received infusions of drugs during counterbalanced test sessions. One half of mice received the degraded session first while other mice received a non-degraded session first. **(B)** Only mice whose cannulae were placed within the IL were included in analyses. Images modified from Paxinos and Franklin (2001). **(C)** Inhibition of D1 signaling with SCH23390 or agonism of the D2 receptor with quinpirole in the IL resulted in reduced responding *only* during the degraded session, consistent with restoration of goal-directed behavior. Error bars ±SEM. ^*^p < 0.05.

Data were square root transformed to maintain homogeneity of variance. To determine whether agonism and/or antagonism of DA D1 or D2 receptors influenced sensitivity to changes in action-outcome relation, active responding during a degraded session was compared to responding during a non-degraded session; during both test sessions the experimental drug was on board. Importantly, no differences in baseline response rates were seen in animals to-be assigned to groups [*F*_(4, 50)_ = 1.122, *p* = 0.356]. Additionally, rmANOVA [drug × non-degraded session (“no drug” vs. “drug”)] revealed no differences were observed in response rates between the “drug” and “no drug” non-degraded session (*p* > 0.5 for main effects, *p* = 0.185 for session × drug interaction). Repeated measures ANOVA revealed a significant session (degraded vs. non-degraded) × drug interaction on active responding [*F*_(4, 46)_ = 2.92, *p* < 0.05]. *Post-hoc* analyses indicated that responding of the saline-injected animals did not differ significantly between the degraded and non-degraded session, indicating that under basal conditions animals were insensitive to the change in action-outcome relations, consistent with the formation of habit. Critically, responding during the degraded session differed significantly from the non-degraded session only for mice receiving the DA D1 receptor antagonist SCH23390 (*p* < 0.05) or the DA D2 receptor agonist quinpirole (*p* < 0.05; Figure 1). Together these data demonstrate that only antagonism of the D1 receptor or agonism of the D2 receptor in the IL are sufficient to restore sensitivity to changes in the action-outcome relationship, indicative of goal-directed instrumental behavior. Mice receiving the DA D1 receptor agonist DHX or DA D2 receptor antagonist sulpiride did not show differential responding between the degraded and non-degraded sessions, confirming that these opposing DA receptor manipulations do not impact sensitivity to changes in contingency after extended training.

Because animals received infusions of the same drug during both test sessions and we used a within subjects analysis to assess responding, we are confident that the marked differences seen between the degraded and non-degraded sessions with either the SCH23390 or the quinpirole infusions reflected a change in response strategy. We do not believe this reduction in responding in the degraded session, which is evidence for goal-directed instrumental action is related to non-specific alterations in task engagement, motivation, or locomotor effects as this would have been reflected as behavioral changes in both the degraded and non-degraded test conditions.

### Compulsive-like food seeking

**Figure 2:**
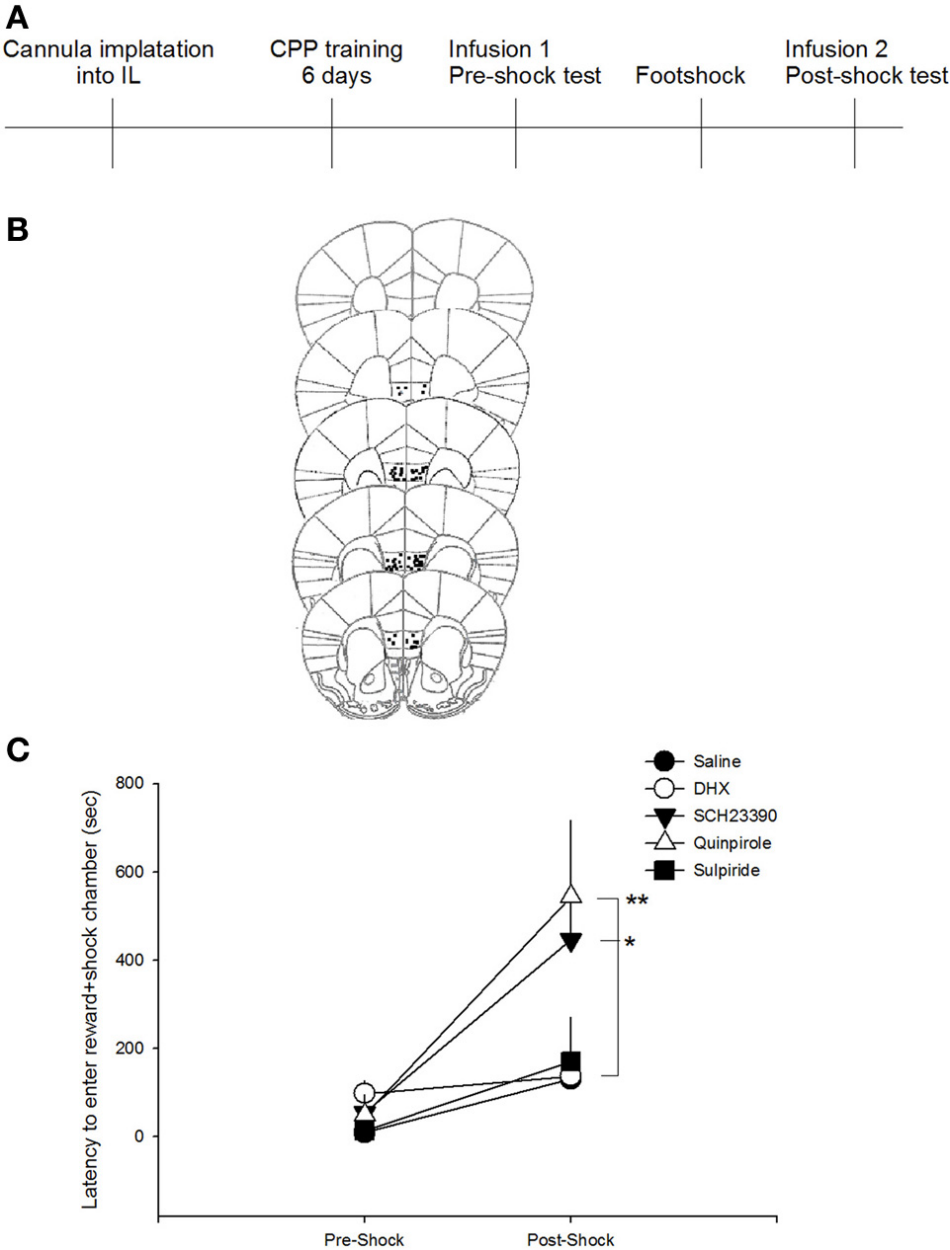
**D1 antagonism or D2 agonism reduce compulsive-like reward seeking. (A)** Experimental timeline. Mice received infusions of drugs into the IL only during test sessions to ensure that effects were on the expression, not acquisition, of compulsive-like behavior. **(B)** Only mice whose cannula could be confirmed to be within the IL were included in behavioral analyses. Images modified from Paxinos and Franklin (2001). **(C)** Antagonism of the D1 receptor with SCH23390 or agonism of the D2 receptor with quinpirole in the IL reduced compulsive-like reward seeking as indicated by an increase in latency to enter the reward-paired chamber *only* after pairing with foot shock (adverse consequence). ^*^p < 0.05, ^**^p < 0.01.

To assess the effect of DA receptor manipulations on a novel measure of compulsive-like reward-seeking behavior, we compared the latency to enter the food reward-paired chamber after training, but prior to shock (pre-shock) with the latency after the animals had received a foot shock in the reward-paired chamber (post-shock). A repeated measures ANOVA revealed a significant session (pre-shock vs. post-shock) × drug interaction on latency to enter the reward paired chamber [*F*_(4, 46)_ = 2.8205, *p* < 0.05]. *Post-hoc* analyses indicated that only animals that received SCH23390 or quinpirole infusions had post-shock latencies that were significantly increased compared to saline-infused animals (*p* < 0.05 and *p* < 0.01, respectively; Figure 2). Neither of these drugs impacted pre-shock latencies, indicating that DA receptor D1 antagonism or D2 agonism increased the latency to enter the reward-paired chamber *only* after that chamber had been paired with a negative consequence. Additionally, administration of quinpirole or SCH23390 did not impact the time spent in the reward-paired chamber in either the pre- or post-shock test [*F*_(2, 21)_ = 0.2022, *p* = 0.8], though there was a main effect of session [*F*_(1, 21)_ = 15.8571, *p* < 0.001]. These data suggest that inhibition of DA D1 or activation of DA D2 receptors do not impact latency to enter the reward paired chamber in situations where there is no conflict, but decrease compulsive-like reward seeking after the risk of aversive outcome has been learned. Post-shock latencies to enter the reward-paired chamber after infusions of DHX or sulpiride, however, did not differ from saline treated mice (*p* > 0.7), indicating that DA D1 agonism or D2 antagonism did not impact compulsive-like reward seeking.

During the pre-shock interval, only mice receiving DHX infusions showed an increase latency to enter the reward paired chamber as compared to saline treated mice (*p* < 0.05), suggesting that DA D1 agonism impacts latency to enter a reward paired chamber under baseline conditions. Again, because mice receive infusions prior to both the pre-shock and post-shock test sessions, we do not think that the ability of SCH23390 or quinpirole to produce increased latencies to enter the post-shock chamber is reflective of altered activity levels or motivation to enter the chamber. To confirm that these manipulations did not generally increase latencies to enter both the paired and unpaired chambers in the post-shock session, a rmANOVA was performed (shock × drug). The analysis revealed a main effect of drug on latency [*F*_(4, 42)_ = 3.26, *p* = 0.02] and a main effect of session [*F*_(1, 42)_ = 5.54, *p* = 0.02], but not shock × drug interaction (*p* = 0.21), suggesting that neither the SCH23390 nor the quinpirole interacted with shock exposure to produce a latency to enter both chambers. Further, these data suggest that exposure to these drugs during the pre-shock session did not result in a generalized aversion to both chambers in the post-shock test session. Follow up analyses indicated that SCH23390 administration resulted in an increased latency to enter the unpaired chamber in both the pre- and post-shock sessions.

## Discussion

These experiments investigated the role of specific manipulations of IL DA D1 and D2 receptor signaling in flexible reward seeking. We found that after extended training in an instrumental task, at a time point when control animals were insensitive to changes in contingency, inactivation of DA D1 or activation of DA D2 receptors in the IL was sufficient to render mice sensitive to the change in the relationship between action and outcome. That is, either a decrease in DA D1 activity or an increase in DA D2 signaling resulted in restoration of goal-directed behavior *after* the transition to habit. Conversely, we saw that neither DA D1 agonism nor DA D2 antagonism had any impact on behavior after extended training, indicating that it is not a general change in the ratio of D1 to D2 signaling that produced this increased sensitivity to action-outcome relationship, but rather specific decreases in DA D1 activity or increases in DA D2 signaling allowed alterations in behavior. Importantly, these studies only investigate one form of loss of action-outcome relationship, and future research will be necessary to determine whether selective infralimbic DA manipulations alter flexible responding in paradigms that disrupt contingency through provision of alternative reinforcers, reversal of the action-outcome contingency through selective reinforcement of non-responding, or under conditions of extinction.

In addition to restoration of goal-directed behavior after extended performance of an instrumental response, we similarly showed that D1 antagonism and D2 agonism in the IL reduced compulsive-like reward seeking in a task investigating competition between adverse consequences and reinforcement. Importantly, we again saw no effects of infralimbic D1 agonism or D2 antagonism on the ability to restore behavioral flexibility. The increase in latency to enter the reward-paired chamber in mice receiving IL infusions of the DA D1 antagonist or DA D2 agonist occurred *only* after animals received a foot shock in the same chamber, indicating that these DA manipulations during the test did not impair either the ability to move toward the chamber or motivation to enter the reward paired chamber in the absence of conflict, i.e., prior to foot shock. Notably, IL DA D2 signaling has been shown to be critical for the extinction of conditioned fear (Mueller et al., 2010). However, we do not think this finding in anyway contradicts our conclusion that IL DA D2 activity reduces compulsive reward seeking as infusion of the DA D2 agonist increases latency to enter the shock and reward-paired chamber, indicating that extinction has not occurred. Together, these data suggest that increased DA signaling through D2-like receptors in the IL restores flexible behavior, while DA D1 activity in the IL may be related to reduced sensitivity to action-outcome relationships, including a loss of such relationships through contingency degradation, and the risk of adverse consequences, as loss of signaling at this receptor restores flexible behavior.

Our lab and several others, have long been interested in the role of corticostriatal dysfunction in inflexible, habitual, addiction-related processes (e.g., Jentsch and Taylor, 1999; Robbins and Everitt, 1999). We have previously demonstrated that administration of exogenous DA into the IL restored goal-directed behavior in animals performing habitually (Hitchcott et al., 2007); our current data suggest that this effect was mediated by activity at DA D2 receptors. Importantly, our current work focuses on the ability of DA manipulations to restore sensitivity in changes to action-outcome contingency, without investigating the role of change in outcome value. While in many cases, response strategy selection in these paradigms is consistent, it is possible that the ability to track action-outcome relationships is dependent on IL DA signaling in a way that is separate from the ability to regulate responding for a devalued outcome, and this has yet to be determined. DA has been shown to differentially affect PFC function depending on the task used and the dose tested. For example, DA is thought to impact measures of prefrontal function, such as working memory, in a dose-dependent manner through D1-mediated alterations in the signal-to-noise ratio (e.g., Arnsten, 2007). Our data indicate that in assessments of habit, exogenous DA is primarily acting through DA D2 receptors to decrease infralimbic activity, which is consistent with the ability of both D2 agonists and DA to restore flexible reward seeking. In addition, this finding reconciles the data from studies indicating that both DA infusions (Hitchcott et al., 2007) and inactivation of the IL restore goal-directed behavior (Coutureau and Killcross, 2003). The activation of DA D1 or D2 receptors has distinct and opposing downstream effects. DA D1 receptors are Gα_s_ coupled, and their stimulation results in increased production of cyclic adenosine monophosphate (cAMP) and the cAMP-dependent protein kinase (PKA). Activation of Gα_*i/o*_ coupled DA D2-like receptors, however, inhibits adenylyl cyclase activity, directly opposing DA D1 activity and downstream signaling. In addition to inhibition of pyramidal cells through the above described mechanism, DA D2 activation may further inhibit projection neurons through enhancement of GABAergic interneuron activity (Tseng and O'Donnell, 2007a). Enhanced signaling at infralimbic DA D2-like receptors relative to D1 receptors is likely to result in decreased neuronal activity. Based on the evidence that inactivation or lesion of the IL also impair the expression of stimulus-response habits (Coutureau and Killcross, 2003; Killcross and Coutureau, 2003), we propose that the ability of DA infusions in the IL to reinstate sensitivity to the action-outcome relationship is due to decreased activity and that the balance of D1/D2 activity in the IL is critical to the expression of flexible reward-seeking behavior.

Though a precise role for infralimbic DA D1 and D2 signaling in habitual and compulsive-like reward seeking has not been previously investigated, IL has been implicated in situations of response conflict (Haddon and Killcross, 2011). Further, a role for prefrontal DA signaling has also been investigated in other measures of flexible behavior. Blockade of DA D1 or D2 in the medial PFC has been shown to impair the ability to update behavior to a change in reward value, while not impacting the ability to perceive the change (Winter et al., 2009). Additionally, DA D2 antagonism impaired flexibility in a set-shifting task, though agonism of DA D2 did not promote shifting (Floresco et al., 2006). Inhibition of the DA D4 receptor, a member of the D2-family of receptors, had opposing effects on set shifting. Consistent with these findings, it is possible that the effects of DA D1 inhibition and D2 activation in our experiments result not from a change in infralimbic activity, but rather through changes in PFC network stability. It has been suggested that DA D1 activity can stabilize the existing PFC networks, potentially explaining why loss of DA D1 signaling can promote flexible behavior through loss of this stabilization (e.g., Seamans and Yang, 2004; Durstewitz and Seamans, 2008). In this model, and consistent with our findings, DA D2 signaling would promote system lability through reduction in signaling in the GABAergic neurons, thus enabling the establishment of new behavioral patterns. The basis for this model, however, is work done in adolescent animals (e.g., Seamans et al., 2001) in which the DA D2 impact on GABAergic signaling may be different (i.e., opposite) from that seen in adult animals (Tseng and O'Donnell, 2007b; O'Donnell, 2010); however, the discrepancy between these findings does not appear to be solely dependent on age (Kroener and Lavin, 2010). It therefore remains unclear whether in adult animals, D2 activation in the IL may act to reduce GABAergic inhibition of pyramidal cells, or perhaps, as described above, to produce a net decrease in IL activity.

As our data suggest that a selective shift in the DA D1:D2 ratio in the IL can enable a shift in response strategy selection, it is important to consider that the observed separation between D1 and D2 effects may result from downstream influences on distinct neuroanatomical targets. It has been well established that in the striatum, DA D1- and D2-receptor containing medium spiny neurons are located in distinct populations of neurons that have separate projection targets. Indeed, striatal D1- and D2-receptor containing neurons that participate in the direct and indirect pathways, respectively, have been shown to differentially contribute to the attribution of value to an action and, therefore, inform response selection in a distinct but complementary fashion (Tai et al., 2012). While there is evidence that PFC neurons may co-express DA D1- and D2-type receptors (Vincent et al., 1995), it has also been demonstrated that D1 and D2 containing neurons are at least in part distinct populations (e.g., Gaspar et al., 1995; Gee et al., 2012). It may be that DA D1- and D2-expressing projection neurons in the IL also have separate targets and that modulation of DA D1 and D2 signaling differentially impacts downstream brain regions, therefore enabling a shift in contribution to response strategy selection between these targets. For example, it has been shown that disconnection of the IL from the nucleus accumbens shell can replicate the effects of IL inactivation on cocaine seeking (Peters et al., 2008). In addition to the nucleus accumbens shell, the IL also projects extensively to amygdalar nuclei (e.g., Vertes, 2004). Though the central nucleus of the amygdala has been shown to interact with the dorsolateral striatum to mediate the expression of goal-directed and habitual behavior (Lingawi and Balleine, 2012), the effect of IL disconnection from its targets on habitual and compulsive reward-seeking behavioral control is still under investigation.

The precise role IL plays in response strategy selection and the mechanism by which decreased activity in the IL would restore goal-directed behavior, remain to be elucidated. Studies by Rich and Shapiro (2009) suggest that infralimbic activity lags behind response switching, while PL activity leads the change, suggesting perhaps that IL is involved in the maintenance of habits while activity in the PL is required to flexibly update responding. Loss of the IL may result in a reversion to the competing memory system that uses knowledge of the action-outcome relationship and outcome value to guide behavior.

### Summary and implications

The ability to behave flexibly is critical to the successful control of reward seeking, and a better understanding of the mechanisms by which response strategies shift away from those that are habitual or compulsive to those that are goal-directed, is likely to inform treatment of both drug and food addiction. Here, we show that increased D2 receptor or decreased D1 receptor activity in the IL can restore sensitivity to changes in action-outcome contingency and decrease reward seeking in the face of punishment. Importantly, these data help to explain the apparent discrepancy between the ability of infusions of DA and inactivation of the IL to enable a shift in response strategy, and will help to inform future work investigating the precise role that IL plays in facilitating plastic behavior.

### Conflict of interest statement

The authors declare that the research was conducted in the absence of any commercial or financial relationships that could be construed as a potential conflict of interest.
